# Effects of Supplemental Feed with Different Levels of Dietary Metabolizable Energy on Growth Performance and Carcass Characteristics of Grazing Naturalized Swan Geese (*Anser cygnoides*)

**DOI:** 10.3390/ani11030711

**Published:** 2021-03-05

**Authors:** Tao Ran, Yi Fang, Hai Xiang, Chengzhen Zhao, Daowei Zhou, Fujiang Hou, Yan D. Niu, Rongzhen Zhong

**Affiliations:** 1 Jilin Provincial Key Laboratory of Grassland Farming, Northeast Institute of Geography and Agroecology, Chinese Academy of Sciences, Changchun 130102, China; rant@lzu.edu.cn (T.R.); fangyi@iga.ac.cn (Y.F.); xianghai@iga.ac.cn (H.X.); zhaochengzhen@iga.ac.cn (C.Z.); zhoudaowei@iga.ac.cn (D.Z.); 2 College of Pastoral Agriculture Science and Technology, Lanzhou University, Lanzhou 730020, China; cyhoufj@lzu.edu.cn; 3 Faculty of Veterinary Medicine, University of Calgary, 2500 University Dr. NW, Calgary, AB T2N 1N4, Canada; dongyan.niu@ucalgary.ca

**Keywords:** grazing versus supplemental feeding, metabolizable energy, growth performance, carcass traits, Swan geese

## Abstract

**Simple Summary:**

Swan geese are becoming a very popular economic wildfowl in China, however, there lacks nutrient requirement guidelines for farmed Swan geese. This study evaluated the effects of supplemental feeding of dietary metabolizable energy (ME) level [9.5, 11.5, and 13.5 MJ/kg of dry matter (DM)] on growth and carcass characteristics of grazing Swan geese. The results showed that growth performance and body-size measurements (except shank length) were greatly improved by offering supplemental feeds to grazing geese, with no difference among supplemental feeding treatments. As DM intake (DMI) decreased with increasing dietary ME from day 29 to 56; meanwhile, slaughter, semi-eviscerated, eviscerated, and thigh muscle yield decreased with increasing dietary ME on day 56, and a well-balanced supplemental diet with ME of 9.5 MJ/kg of DM was recommended to improve growth and meat production of grazing Swan geese.

**Abstract:**

Grazing Swan geese (*Anser cygnoides*) have good meat quality but grow slowly. This study aimed to study whether supplemental feeding could improve growth performance of grazing Swan geese and investigate a suitable dietary metabolizable energy (ME) level of supplemental diet for grazing Swan geese. Naturalized healthy male Swan geese (*n* = 144; 42 ± 2.0 days and 1.21 ± 0.17 kg) were randomly allocated into 4 groups and grazed on pasture alone (control, CON) or offered supplemental diets with ME of 9.5, 11.5, or 13.5 MJ/kg of DM after grazing. Growth performance and body-size measurements (including bone development) were lower (*p* < 0.05) in CON versus supplemented geese, as well as slaughter measurements on days 28 and 56. The DM intake linearly decreased (*p* < 0.01) with increasing dietary ME from day 29 to 56. Slaughter, semi-eviscerated, eviscerated, and thigh muscle yield linearly (*p* < 0.01) decreased with increasing dietary ME on day 56. Lightness (L*) and yellowness (b*) for breast and thigh muscle on days 28 and 56, and breast muscle shear force on day 56, were lower (*p* < 0.01) in supplemented versus CON geese. In conclusion, supplemental feeding improved growth performance and carcass characteristics of grazing Swan geese, and supplemental feed with ME of 9.5 MJ/kg of DM could be offered to improve growth and meat quality of grazing Swan geese.

## 1. Introduction

Swan geese (*Anser cygnoides*) are migratory birds that are widely distributed in Mongolia, northeastern China, and southeastern Russia. Compared to intensively produced domestic geese, Swan geese meat is considered healthier due to its high protein but low fat and cholesterol [1]. Thus, making it one of the most popular and economical wildfowl in several provinces of China. Consequently, there is increasing interest in raising naturalized Swan geese. However, there is no published nutrient requirements for farmed Swan geese, and its feed formulation is usually based on requirements for domestic geese or ducks.

Farmed Swan geese are commonly reared in artificial environments like domestic geese, eating forages and/or formulated diets until they reach a standard market body weight (BW) of 3.0–3.5 kg. There are some common concerns, such as (1) will geese reach market BW on time if fed only forage? (2) Will grazing pasture alone have negative effects on bone development and will it affect fattening performance? (3) If geese are fed only formulated diets, will they be over-fattened and have lower meat quality? These concerns are also relevant for domestic goose production. Domestic geese fed only forages cannot reach standard market BW by 70 days of age [2]. Furthermore, early nutrition affects growth rate and physical development of birds, especially adult skeletal size [3,4]. Excessive fat accumulation, especially abdominal fat, is a major problem facing modern commercial poultry production, leading to reduced growth efficiency and meat quality [5]. Therefore, determining a feeding regime that optimizes both growth efficiency and meat quality of Swan geese is needed.

Dietary composition and feeding regimes have potential to improve growth performance, promote physical development, and reduce body fat deposition in poultry [6]. Regulating dietary metabolizable energy (ME) is one of the most common strategies, and its effects on growth performance and carcass characteristics have been studied in domestic ducks and geese [7,8,9], with ME of 10.87–11.29 MJ/kg and 11.29–12.13 MJ/kg recommended for geese of 0–4 and 5–10 weeks of age [10], respectively. However, relevance of these recommendations for farmed Swan geese are unknown. Moreover, effects of feeding management, e.g., intensive feeding versus free range feeding on growth performance, carcass traits, and meat quality, were studied for domestic geese [2,11,12,13], but apparently not for Swan geese.

We hypothesized that offering grazing Swan geese with supplemental feed containing suitable dietary ME would promote growth performance and physical development and improve carcass traits and meat quality without adversely affecting health. Therefore, our objective was to investigate effects of offering supplementary feeding diets with various levels of ME on growth performance, physical and bone development, carcass characteristics, and blood biochemistry of grazing naturalized Swan geese (*Anser cygnoides*).

## 2. Materials and Methods

### 2.1. Animals and Experiment Design

This study was conducted on a pasture at the Changling Ecological Research Station for Grassland Farming at the Songnen Plain in the Northeast of China (44°33′ N, 123°31′ E; 145 m above sea level), where annual average temperature is 4.9 to 6.4 °C and annual average precipitation ranges from 250 to 500 mm, with 70–80% of total precipitation from June to September. Healthy, naturalized Swan geese (*Anser cygnoides*; *n* = 144, 42 ± 2.1 days and 1.21 ± 0.17 kg) grown under artificial feeding conditions were provided by Benjun Geese Specialized Farming Cooperative Society (Jilin, China). All geese were given 1 week to adapt to the new environment and forages before experiment. During adaptation, each goose was labeled with a leg ring and vaccinated following standard operating procedures. At the end of adaptation, all geese were blocked by BW and randomly allocated into 4 groups (6 replicates per group and 6 geese per replicate), which were then randomly allocated to 1 of 4 treatments: grazed on pasture alone (control, CON) or offered supplemental diets with low, medium, or high ME (9.5, 11.5, and 13.5 MJ/kg of dry matter (DM); LMED, MMED, and HMED, respectively) after grazing. The diets were formulated to meet nutrient requirements of geese [10,14], with minor modifications (Table 1). The grazing pasture was composed of perennial *Leymus chinensis, Chloris virgate,* and *Puccinellia tenuiflora* (approximately 70.5%, 18.2%, and 8.4%, respectively), with a small amount of leguminous grass.

### 2.2. Feeding Management, Sampling, and Measurements

During the study, geese were let out for grazing pasture at 06:00 am and returned to the pen at 17:00 pm, with 6 geese in each replicate housed in the same pen at night. For the 3 supplemental feeding treatments, supplemental diets were offered ad libitum by pen at 18:00 pm, with refusals weighed before the next feeding. All geese had free access to clean water. The daily supplemental DM intake (DMI) was calculated by pen as (daily DM offered–daily DM refused)/6. Geese were weighed on days 0, 28, and 56 and average daily gain (ADG) was calculated for each 28-day period and the overall experiment period. Samples of diets were collected weekly and pooled over the entire experimental period, mixed, subsampled, dried in an oven at 55 °C for 48 h, then ground through a 1 mm screen and stored. All chemical analyses including ME, DM, crude protein (CP), and crude fiber were done as described previously [15]. Calcium was determined by atomic absorption spectrophotometry and phosphorus was estimated colorimetrically, as described previously [16].

### 2.3. Blood Sampling and Slaughter

Two geese of each replicate (with average BW) were selected for blood sampling and slaughter at 09:00 am on days 0, 28, and 56. Blood sample (~5 mL) was collected via wing root venipuncture using a 7 mL vacuum tube without additive (Vacutainer, Becton Dickinson, Franklin Lakes, NJ) at each sampling time. Blood samples were kept at 4 °C for 30 min, then centrifuged at 2000× *g* for 15 min at 4 °C and frozen at −20 °C until analyzed. Serum biochemical parameters, including albumin (ALB), total protein (TP), triglyceride (TG), total cholesterol (TC), blood urea N (BUN), and alkaline phosphatase (ALP), were determined using commercial kits (Jiancheng Biology Co., Nanjing, China) and an automated biochemical analyzer (7020 Model, Hitachi, Beijing, China). Serum globulin (GLO) content was calculated as the difference between TP and ALB.

After blood sampling, geese were exsanguinated by severing the jugular vein and carotid artery on one side of the neck. Weight of each goose was recorded after bleeding and then measurements of body-size (body length, chest width, chest depth, keel length, tibia, hip width, and neck length) were determined. Thereafter, geese were plucked and eviscerated. Measurements of carcass traits (including weights of semi-eviscerated carcass, eviscerated carcass, and subcutaneous fat, and meat quality of thigh muscle and breast muscle), length of various sections of digestive tract (including duodenum, jejunoileal, cecum, and rectum), and weights of viscera (including heart, liver, kidney, lung, spleen, pancreas, gizzard, and proventriculus) were recorded. Slaughter yield, semi-eviscerated carcass yield, eviscerated yield, and relative weights of viscera were calculated as percentage of live BW, whereas yield of subcutaneous fat, breast muscle, and thigh muscle were calculated as percentage of eviscerated carcass weight. Meat quality was assessed based on breast muscle color and shear force. The meat color, where L* represents lightness, a* redness, and b* yellowness, was measured using a Minolta CR-410 color-meter (Minolta Camera Co., Ltd., Osaka, Japan). Shear force analysis was conducted according to guidelines of the American Meat Association [17], using a Warner-Bratzler Shear Device (C-LM3B, Beijing, China).

### 2.4. Statistical Analyses

All data were analyzed using the Mixed procedure of SAS (version 9.1, SAS Institute Inc., Cary, NC, USA) as a completely randomized design to account for time (days on feed) effects, with treatment, period (weigh day), and geese within treatment as random effect. For repeated measures, various covariance structures were tested, and Autoregressive (1) was selected based on the lowest value for Akaike’s information criteria. To test the effects of increasing dietary ME level on measured variables, orthogonal contrasts were conducted with unequal treatment spacing orthogonal polynomial contrast coefficients calculated to determine linear and quadratic responses. Least square means were compared using the Tukey correction for multiple comparisons and treatment effects were declared significant at *p* ≤ 0.05 and trends at 0.05 < *p* ≤ 0.10.

## 3. Results

### 3.1. Growth Performance and Body-Size Measurements

The BW (days 28 and 56) and ADG (days 0–28, days 29–56, and overall) of supplemented geese were greater (*p* < 0.05) than CON geese, but there were no significant differences in BW and ADG among the 3 supplemented treatments (Table 2). Notably, ADG of grazing geese increased (*p* < 0.01) from 9.40 g/day to 20.55 g/day, whereas ADG of supplemental feeding decreased (*p* < 0.01) from 40.65 g/day (averaged) to 26.12 g/day during days 29 to 56. The DMI was not different among LMED, MMED, and HMED treatments from day 0 to 28, but linearly decreased (*p* < 0.01) as levels of dietary ME increased from day 29 to 56; therefore, overall DMI linearly decreased (*p* < 0.01) with increasing dietary ME. There were effects of days on-feed for BW, DMI, and ADG (*p* < 0.01; Table 2).

Most body-size measurements were greatly enhanced (*p* < 0.05) by supplemental feeding as compared to grazing pasture only (Table 3), with minimal differences among supplemented groups. There was an effect of days on-feed (*p* < 0.05) for all body-size measurements (Table 3).

### 3.2. Carcass Traits and Meat Quality

On both days 28 and 56, greater (*p* < 0.01) slaughter, semi-eviscerated, and eviscerated weights were observed in supplemented versus grazed geese, with no differences among supplemented groups (Table 4). Slaughter yield was not different among treatments, except lower (*p* < 0.03) slaughter yield for geese of HMED on day 28 and slaughter yield linearly (*p* < 0.01) decreased with increasing dietary ME on day 56. Similarly, the semi-eviscerated yield did not differ among treatments, except greater semi-eviscerated yield for geese of LMED on day 28; furthermore, there was a linear (*p* < 0.01) decrease in semi-eviscerated yield with increasing dietary ME on both days 28 and 56. However, there was greater (*p* < 0.05) eviscerated yield in MMED and HMED than CON geese on day 28 and in LMED and MMED than CON geese on day 56, with a linear (*p* < 0.01) decrease of eviscerated yield with increasing dietary ME. Abdominal fat yield did not differ among treatments on day 28, but it was greatly (*p* < 0.01) increased in supplemented versus control geese on day 56. Thigh muscle yield was greatly (*p* < 0.01) enhanced by supplemental feeding as compared to the grazing geese on both days 28 and 56, and it increased linearly (*p* < 0.02) with increasing dietary ME. Breast muscle yield was not affected by treatment on day 28, but CON geese had greater (*p* < 0.01) breast muscle yield on day 56 than supplemented geese. There were effects (*p* < 0.05) of days on-feed for all carcass trait measurements (Table 4).

Values of L*, a*, and b*, and shear-force of breast muscle were affected (*p* < 0.01) by treatment, except a* value on day 56 (Table 5). The L* and b* values of breast muscle were lower (*p* < 0.01) in supplemented versus CON geese on both days 28 and 56, with no difference among LMED, MMED, and HMED. The a* of breast muscle was higher (*p* < 0.01) in supplemented versus CON geese on day 28, however, the difference was eliminated on day 56. Shear force of breast muscle of geese of LMED treatment was significantly lower (*p* < 0.01) than the other three treatments on day 28, whereas shear force of breast muscle of CON geese was significantly greater (*p* < 0.01) than that of supplemented geese on day 56. There were effects (*p* < 0.01) of days on-feed for L*, a*, b*, and shear force (Table 5).

### 3.3. Length of Gut Section and Relative Weights of Viscera

The only effect (*p* < 0.05) of treatment on length of digestive tract segments was jejunoileal segment of CON versus supplemented geese on day 56 (Table 6). There were effects of days on-feed (*p* < 0.05) for all sections.

Relative weights of heart and spleen were not different among groups on both days 28 and 56 (Table 7). Relative weights of liver were affected (*p* < 0.01) by feeding management, with greater relative weight of liver in LMED and MMED geese than CON and HMED geese on day 28, and with greater value in grazing geese than in supplemental feeding geese on day 56. Furthermore, relative weight of liver linearly decreased (*p* < 0.04) with increasing levels of dietary ME on both days 28 and 56. Relative weights of kidney and spleen were not different among treatments on day 28, whereas it was lower (*p* < 0.01) for supplemented geese on day 56. Supplemented geese had lower (*p* < 0.01) relative weights of pancreas, gizzard, and proventriculus than CON geese on both days 28 and 56, and relative weight of proventriculus linearly (*p* < 0.02) decreased with increasing levels of dietary ME on day 28, as well as for relative weights of pancreas (*p* < 0.01) and gizzard (*p* < 0.05) on day 56.

### 3.4. Serum Biochemical Parameters

Serum biochemical parameters were differentially affected by treatments on days 28 and 56, with all serum biochemical parameters except glucose affected by treatments on day 28, whereas TG, TC, and ALP were not affected by treatments on day 56 (Table 8). On day 28, serum ALB, GLO, and TP were not different among CON, LMED, and MMED treatments, which were greater (*p* < 0.05) than that of HMED geese. Serum TC was greater (*p* < 0.01) in geese that grazed pasture only than in geese that received supplemental feeding, serum ALP was greater (*p* < 0.01) in LMED and MMED geese than in CON and HMED geese, and serum ALB, GLO, TP, TG, TC, and ALP linearly (*p* < 0.05) decreased with increasing levels of dietary ME. However, serum ALB, GLO, TP, BUN, and glucose in CON geese were lower (*p* < 0.01) than that in geese of MMED and HMED on day 56. Furthermore, all these measurements linearly (*p* < 0.02) increased with increasing levels of dietary ME.

## 4. Discussion

Geese can consume green forages and fibrous byproducts of crops to derive a considerable amount of their nutrient requirements [18]. Dietary fiber is essential for geese to maintain normal performance; consequently, a low-fiber diet had negative effects on nutrient utilization and health, with poor growth and slaughter performance [19]. Although wild geese can be grown entirely on pasture and domestic geese also can subsist almost entirely on forage, duration of feeding prior to reaching market weight was prolonged by 2–3 weeks [14]. Domestic geese that consumed forage failed to reach standard market weight (3.0–3.5 kg) on time (usually 120 days of age) due to inadequate CP and energy in forage [2]. In the current study, Swan geese that grazed on pasture alone for 56 days had a final BW of 1932.1 g, which were well below standard market weight. In contrast, grazed Swan geese that received supplemental feeding had a final BW of ranging from 3008.3 to 3258.3 g, meeting the target for standard market weight. The lower final BW of CON geese was attributed to their lower feed intake as compared to supplemented geese, which was expected, as nutrients derived from pasture may not fulfill nutrient requirements. Current results were consistent with previous studies [2,11,12], in which grazing geese had significantly lower BW than those fed supplemental feeds. Furthermore, all body-size measurements, except shank length, were significantly lower in CON versus supplemented geese, suggesting that bone development was slowed due to inadequate macronutrients. There were significantly lower body-size parameters in grazing geese and grazing geese that received only 1 week of supplemental feeding at the end of fattening compared to those with supplemental feeding throughout the experimental period [2]. Therefore, we recommended that supplementing grazing Swan geese is a practical method to obtain ideal growth performance by providing well-balanced nutrition. This is not only a feasible method to ensure geese reach standard market weight by 100 days, but also to promote bone development.

It was noteworthy that BW of geese supplemented with diets with ME ranging from 9.5 to 13.5 MJ/kg were not different throughout the study. Similarly, weight gain of Italian Legarth goslings on starter and grower diets with ME ranging from 11 to 13 MJ/kg did not differ significantly [16]. In addition, similar results were also reported in ducks with dietary ME ranging from 11.8 to 13.8 MJ/kg [9]. This was attributed to decreased feed intake as energy concentration increased, thereby achieving consistent energy intake [9,16]. Furthermore, excess energy consumption does not further increase body weight gain but makes apparent digestibility of nutrients decline [20]. Notably, ADG of supplemented geese decreased dramatically from day 29 to 56, accompanied by decreased DMI as compared to days 0 to 28. Similarly, there was a very rapid live weight gain of goslings during the starter period, followed by a gradually reduced live weight gain after 10 weeks of age, with poor feed conversion [21]. Although geese are fast growing, their efficiency to convert feed to weight gain diminished rapidly with age [16]. Despite compensatory growth for grazing geese during days 29 to 56, their bone development and growth was greatly reduced due to low nutrient quality and quantity. Offering supplemental feeding to grazing geese at the end of the fattening period for only 1 week (days 64 to 70) did not enable geese to reach standard market weight, as it was too late to support optimal bone development [2]. Geese have reached complete bone development after 8 weeks, whereas development of breast muscle continues until 9 to 10 weeks [22]. Thus, it is critical to offer supplemental feeding at the proper time to avoid irreversibly retarded bone development.

Geese have a stronger gizzard than other waterfowl, making them successful grazers, able to break down and digest plant cell walls [14,23]. As degradation in the gizzard is largely mechanical, geese that accessed grazing pasture or were supplemented with roughage always had higher relative weights of gizzard and a longer digestive tract. In the current study, there were significantly greater relative weights of gizzard and proventriculus in CON geese on days 28 and 56, as well as a longer jejunoileal segment on day 56. Due to lower nutrient contents and poorer feed efficiency of pasture, grazing geese had to ingest more pasture than supplemented geese to meet nutrient requirements. Greater roughage intake increased bulk of digesta, which triggers increasing contraction frequency and weight of gizzard and proventriculus to promote digestion. Geese fed >20% defatted rice bran had greatly increased proventriculus weights [24]. Increased jejunoileal length of grazing geese implies increased intestinal surface area for nutrient absorption. It is accepted that geese modify volume and weight of digestive tract and digesta passage rate to adapt to fiber-rich diets [25]. However, there are also some contradictory reports that fiber-rich diets shortened relative lengths of duodenum, jejunum, and ileum [26,27], or had no effects on length and weight of gastrointestinal tract sections [21]. Apparent discrepancies in relative lengths of the digestive tract were possibly caused by breed and age and by sources and levels of fiber [26,27], and perhaps other factors, e.g., physical form of diets [28,29]. Similar relative weights of gizzard and proventriculus and length of various sections of intestine of geese that received diets with varying dietary ME, implies similar digestion and absorption ability, consistent with their similar growth performance and body-size measurements.

Carcass traits, reflecting differential deposition of nutrients in various tissues or different parts of the same tissue, are important indicators in evaluating growth performance of meat animals [24]. In the current study, supplemented geese had significantly greater slaughter weight, semi-eviscerated weight, and eviscerated weight than CON, which was consistent with several previous studies. For example, geese under intensive or semi-intensive feeding had significantly greater carcass weight and edible meat production than those under a pasture system [12]. Supplemented geese had significantly greater eviscerated carcass yield than grazed geese [2], whereas intensively reared geese had higher slaughter and carcass part weights than free-range geese [30]. In the present study, geese that grazed pasture alone had significantly lower thigh muscle yield than supplemented geese, in direct contrast to a previous report [2], which was perhaps due to differences in animal management that altered animal movements and exercise. In that regard, supplemented geese were grazed during the day in the present study but kept indoors in the previous study [2]. Perhaps, when geese are fed a well-balanced diet, more exercise may promote thigh yield. Poorer carcass quality of grazing geese was mainly due to a higher dietary fiber intake and lower feed digestibility, plus unbalanced and inadequate available nutrients [12]. Notwithstanding, based on previous studies [2,12,30], grazing geese have some advantages to intensively fed geese, e.g., less subcutaneous and abdominal fat, lower percentage of skin, and greater meat quality. Similarly, in the present study, there was significantly lower abdominal fat yield in CON versus supplemented geese, which agrees with Liu and Zhou [31] that pasture intake reduced subcutaneous fat thickness and abdominal fat yield of geese compared to control. As the main organ involved in fat metabolism, relative liver weights were significantly lower in grazing versus supplemented geese on day 28, consistent with El-Hanoun et al. [12]. However, greater relative weights of liver in grazing geese were probably due to a compensatory response to low dietary fat level, as reported [7]. Furthermore, breast and thigh muscles of grazing geese had significantly higher protein content and muscle collagen than those of intensively fed geese [2]. These advantages not only increase consumer demand, but also stimulate producers to seek ways to improve both meat quality and growth rate. Either supplemental feeding of grazing geese or adding an appropriate portion of forage to intensively fed geese were effective to modulate meat quality and growth rate. Liu and Zhou reported improved carcass characteristics, meat quality, and enhanced polyunsaturated fatty acid ratios in geese with ad libitum access to a corn-based ration and an alfalfa-based pasture [31]. Janicki et al. also reported that semi-intensive feeding resulted in lesser monounsaturated and higher polyunsaturated fatty acids in abdominal fat [11]. Greater meat L* and b* in CON geese was in line with previous studies of higher breast meat b* and thigh meat L* values in poultry reared in a free-range system [23,30,32]. Shear force is used for evaluating tenderness of meat (samples with lower shear force are more tender). In the current study, supplemented geese had significantly lower shear force than CON geese on day 56, suggesting that supplemental feeding will increase meat tenderness of grazing Swan geese, presumably due to increased intermuscular fat.

Although slaughter, semi-eviscerated, and eviscerated weights did not differ among geese offered diets with ME ranging from 9.5 to 13.5 MJ/kg, slaughter, semi-eviscerated, and eviscerated yields linearly decreased with increasing dietary ME, as well as a tendency for increased abdominal fat yield. Similarly, in a previous study, dietary ME significant affected eviscerated yield, which peaked at 11.87 MJ of ME/kg of diet [7]. Current results concerning abdominal fat yield also agreed with previous reports in both geese and ducks that increasing dietary ME was associated with increased abdominal fat percentage [7,8,9], whereas relative abdominal fat weight was reduced significantly by decreasing dietary ME in broiler chickens, ducks, and geese [5].

Health status can be reflected by serum biochemical parameters. Serum cholesterol, triglyceride, and total protein concentrations were lowered by feeding fibrous diets [21]. In the current study, serum ALB, GLO, and TP did not differ between CON geese and those supplemented with LMED or MMED diets on day 28, whereas they were significantly lower in grazing Swan geese than all the supplemented geese on day 56. This was likely attributed to an insufficient CP intake of geese that grazed pasture alone as compared to geese supplemented with well-balanced diets. Similarly, replacing a basal diet with 20% grass meal or dried sugar beet pulp meal during the grower period significantly decreased serum TP and ALB concentrations [21]. Greater BUN and glucose in geese supplementally fed MMED and HMED diets than the grazing geese, suggested better N and energy metabolism status in supplemental fed geese. Numerically greater TC in supplemental fed geese than the grazing geese on day 56 agreed with results of abdominal fat yield. However, although the value fluctuated from day 0 to 56 and among treatments, that all serum biochemical parameters were within a normal range, based on a previous report [24], suggests that all geese were under good health during the study.

## 5. Conclusions

In conclusion, offering a supplemental diet of well-balanced nutrients to grazing geese at the grower stage not only significantly promoted growth and bone development of Swan geese, but also produced more edible meat without negative effects on meat quality. Thus, it is a feasible and economical practice for farmed Swan geese production. Most end points of growth performance, body-size, carcass trait, and meat quality, did not differ among geese fed any of the three supplemental diets. However, considering that slaughter, semi-eviscerated, eviscerated, and thigh muscle yields linearly decreased while abdominal fat yield linearly increased with increasing dietary ME, we concluded that ME of supplemental diet should be <9.5 MJ/kg of DM to optimize growth performance and meat quality.

## Figures and Tables

**Table 1:** Ingredients and chemical compositions of 3 diets for supplementing grazing Swan geese.

Item	Groups ^†^		
	LMED	MMED	HMED
Ingredient (% of DM)			
Ground corn	64.0	62.0	60.0
Soybean oil	0	3.5	7.0
Soybean meal	15.0	17.0	18.0
Fish meal	1.0	3.0	5.0
Wheat bran	16.5	10.5	6.0
Calcium hydrogen phosphate	1.35	1.35	1.35
Limestone	1.0	1.0	1.0
Sodium chloride	0.3	0.3	0.3
L-methionine + Cystine	0.2	0.2	0.2
Carnitine	0.15	0.15	0.15
Minerals and vitamins salt ^‡^	1.0	1.0	1.0
Chemical composition			
ME (MJ/kg of DM) ^§^	9.5	11.5	13.5
ME (MJ/kg of DM) ^¶^	9.3	11.2	12.9
DM (% of feed)	92.5	92.4	92.2
Crude protein (% of DM)	14.6	15.2	15.9
Crude fiber (% of DM)	7.8	7.3	7.1
Available P (% of DM)	0.41	0.43	0.41
Ca (% of DM)	0.75	0.75	0.75

^†^ LMED: low metabolizable (ME) diet [9.5 MJ/kg of dry matter (DM)]; MMED: medium ME diet (11.5 MJ/kg of DM); HMED: high ME diet (13.5 MJ/kg of DM); ^‡^ Contained (per kg): 5,000,000 international unit (IU) vitamin A, 500,000 IU vitamin D, 100,000 IU Vitamin E, 8 mg Vitamin K, 5 mg vitamin B1, 14 mg vitamin B2, 5 mg vitamin B6, 20 μg vitamin B12, 38 mg niacin, 9 mg pantothenic acid, 1 mg folic acid, 35 μg biotin, 6 g choline chloride, 5 mg Cu, 100 mg Fe, 110 mg Zn, 100 mg Mn, 0.15 mg Se, and 0.5 mg Co; ^§^ Calculated value; ^¶^ Determined value.

**Table 2:** Effects of supplemental feeding diets with three levels of metabolizable energy (ME) on growth performance of grazing Swan geese.

Items	Groups ^†^				SEM ^§^	*p*-Value ^‡^<		
	CON	LMED	MMED	HMED		Treat	Linear	Quadratic
BW (g)								
day 0	1260.2	1257.4	1259.8	1256.3	48.96	1.00	0.89	0.65
day 28	1523.6 ^b^	2275.0 ^a^	2491.7 ^a^	2421.6 ^a^	90.99	0.01	0.33	0.27
day 56	1932.1 ^b^	3008.3 ^a^	3258.3 ^a^	3116.7 ^a^	92.22	0.01	0.48	0.15
DMI ^§^ (g/day)								
day 0–28	NA	127.22	127.78	126.91	0.456	0.22	0.52	0.11
day 29–56	NA	102.03 ^a^	99.20 ^b^	97.02 ^b^	1.010	0.05	0.01	0.79
overall	NA	114.63 ^a^	113.49 ^a,b^	111.96 ^b^	0.578	0.05	0.01	0.76
ADG (g/day)								
day 0–28	9.40 ^b^	36.33 ^a^	44.00 ^a^	41.62 ^a^	3.298	0.01	0.34	0.29
day 29–56	20.55 ^b^	26.18 ^a^	27.37 ^a^	24.82 ^a,b^	1.997	0.05	0.67	0.51
overall	14.97 ^b^	31.23 ^a^	35.70 ^a^	33.23 ^a^	1.587	0.01	0.44	0.14

^a,b^ Within a row, least square means without a common superscript differed (*p* < 0.05). ^†^ LMED: low ME diet (9.5 MJ/kg of DM); MMED: medium ME diet (11.5 MJ/kg of DM); HMED: high ME diet (13.5 MJ/kg of DM); ^§^ Standard Error of Mean; ^‡^ Treat represents *p*-value of four treatments; Linear and Quadratic represents *p*-value of linear and quadratic change of supplemental feeding treatments (LMED, MMED, and HMED), respectively; ^§^ DMI represented only for supplemental feeding diets, as it was impossible to measure pasture intake during grazing; NA: not available; CON: control.

**Table 3:** Effects of supplemental feeding diets with three levels of metabolizable energy (ME) on body-size measurements of grazing Swan geese.

Slaughter Day	Body-Size Measurement	Groups ^†^				SEM ^§^	*p*-Value ^‡^<		
		CON	LMED	MMED	HMED		Treat	Linear	Quadratic
0	Body length (cm)	20.67	20.28	20.24	20.26	0.276	0.41	0.95	0.91
	Chest width (mm)	72.94	72.66	72.56	73.54	0.982	0.90	0.44	0.58
	Chest depth (mm)	79.32	78.21	75.67	77.40	1.615	0.38	0.70	0.26
	Keel length (mm)	95.12	95.84	94.71	95.94	1.302	0.88	0.96	0.48
	Shank length (mm)	80.32	88.44	80.79	83.74	3.670	0.29	0.53	0.05
	Tibia (cm)	4.18	4.25	4.17	4.18	0.049	0.58	0.28	0.45
	Hip width (cm)	7.74	7.90	7.89	7.95	0.054	0.62	0.70	0.79
	Neck length (cm)	18.82	18.43	18.57	18.72	0.294	0.79	0.47	0.97
28	Body length (cm)	21.83 ^b^	24.23 ^a^	25.12 ^a^	24.95 ^a^	0.590	0.01	0.43	0.50
	Chest width (mm)	55.90 ^b^	73.66 ^a^	74.84 ^a^	80.09 ^a^	2.723	0.01	0.04	0.41
	Chest depth (mm)	83.54 ^b^	92.43 ^a^	93.29 ^a^	97.56 ^a^	2.858	0.02	0.23	0.64
	Keel length (mm)	96.06 ^b^	121.08 ^a^	122.36 ^a^	122.71 ^a^	4.552	0.01	0.80	0.93
	Shank length (mm)	84.90	86.91	88.37	89.21	2.368	0.60	0.55	0.93
	Tibia (cm)	4.12 ^b^	4.65 ^a^	4.44 ^a^	4.48 ^a^	0.110	0.02	0.29	0.36
	Hip width (cm)	64.93 ^b^	78.37 ^a^	77.06 ^a^	80.35 ^a^	2.828	0.01	0.47	0.34
	Neck length (cm)	18.60	19.85	20.77	20.45	0.877	0.27	0.61	0.54
56	Body length (cm)	25.29 ^b^	28.22 ^a^	28.07 ^a^	27.85 ^a^	0.548	0.01	0.64	0.96
	Chest width (mm)	75.26 ^b^	83.52 ^a^	90.10 ^a^	85.42 ^a^	2.736	0.01	0.63	0.11
	Chest depth (mm)	99.14 ^b^	110.47 ^a^	112.68 ^a^	105.08 ^a,b^	3.552	0.02	0.26	0.24
	Keel length (mm)	119.43 ^b^	149.38 ^a^	151.33 ^a^	143.09 ^a^	5.013	0.01	0.40	0.43
	Shank length (mm)	83.35	86.60	87.82	85.96	1.759	0.35	0.82	0.53
	Tibia (cm)	4.28 ^c^	4.72 ^a,b^	4.99 ^a^	4.56 ^b^	0.096	0.01	0.28	0.01
	Hip width (cm)	72.22 ^b^	87.20 ^a^	86.48 ^a^	79.80 ^a,b^	3.733	0.04	0.22	0.56
	Neck length (cm)	21.39 ^b^	23.54 ^a,b^	24.43 ^a^	24.39 ^a^	0.806	0.05	0.50	0.67

^a–c^ Within a row, least square means without a common superscript differed (*p* < 0.05). ^†^ CON: control; LMED: low ME diet (9.5 MJ/kg of DM); MMED: medium ME diet (11.5 MJ/kg of DM); HMED: high ME diet (13.5 MJ/kg of DM); days on-feed was *p* < 0.05 for all items; ^§^ Standard Error of Mean; ^‡^ Treat represents *p*-value of 4 treatments; Linear and Quadratic represents *p*-value of linear and quadratic change of supplemental feeding treatments (LMED, MMED, and HMED), respectively.

**Table 4:** Effects of supplemental feeding of three levels of metabolizable energy (ME) on carcass traits of grazing Swan geese.

Slaughter Day	Carcass Trait	Groups ^†^				SEM *	*p*-Value ^‡^<		
		CON	LMED	MMED	HMED		Treat	Linear	Quadratic
0	Slaughter weight (g)	1071.6	1090.4	1072.2	1100.6	42.58	0.30	0.45	0.06
	Semi-eviscerated weight (g)	966.7	955.2	971.4	968.9	37.73	0.59	0.23	0.34
	Eviscerated weight (g)	820.0	847.1	829.2	813.5	35.43	0.18	0.05	0.93
	Slaughter yield ^§^ (%)	84.87	86.88	85.38	87.64	0.833	0.11	0.48	0.07
	Semi-eviscerated yield ^§^ (%)	76.68	76.10	77.23	77.22	0.818	0.41	0.20	0.45
	Eviscerated yield ^§^ (%)	64.97	67.40	65.96	64.72	1.752	0.39	0.01	0.90
	Abdominal fat yield ^¶^ (%)	0.23	0.23	0.19	0.25	0.031	0.43	0.62	0.08
	Thigh muscle yield ^¶^ (%)	8.86	8.63	8.02	8.13	0.437	0.44	0.27	0.36
	Breast muscle yield ^¶^ (%)	/	/	/	/	/	/		
28	Slaughter weight (g)	1272.0 ^b^	2075.9 ^a^	2149.6 ^a^	2141.9 ^a^	104.13	0.01	0.69	0.77
	Semi-eviscerated weight (g)	1141.0 ^b^	1906.1 ^a^	1916.1 ^a^	1941.2 ^a^	95.83	0.01	0.81	0.95
	Eviscerated weight (g)	941.7 ^b^	1549.2 ^a^	1667.1 ^a^	1696.5 ^a^	89.83	0.01	0.29	0.71
	Slaughter yield ^§^ (%)	89.73	91.29	91.91	88.68	1.100	0.18	0.12	0.18
	Semi-eviscerated yield ^§^ (%)	80.41 ^b^	84.04 ^a^	81.49 ^b^	80.19 ^b^	0.776	0.01	0.01	0.55
	Eviscerated yield ^§^ (%)	66.26 ^b^	68.47 ^a,b^	70.91 ^a^	69.98 ^a^	1.142	0.04	0.41	0.29
	Abdominal fat yield ^¶^ (%)	0.27	0.22	0.34	0.31	0.043	0.25	0.14	0.16
	Thigh muscle yield ^¶^ (%)	1.55 ^c^	3.69 ^b^	4.05 ^a,b^	4.64 ^a^	0.329	0.01	0.05	0.77
	Breast muscle yield ^¶^ (%)	6.20	6.90	6.21	6.23	0.334	0.39	0.15	0.38
56	Slaughter weight (g)	1679.1 ^b^	2676.0 ^a^	2834.5 ^a^	2638.5 ^a^	90.96	0.01	0.78	0.13
	Semi-eviscerated weight (g)	1528.0 ^b^	2451.3 ^a^	2608.2 ^a^	2437.6 ^a^	86.63	0.01	0.91	0.15
	Eviscerated weight (g)	1298.4 ^b^	2189.7 ^a^	2324.8 ^a^	2143.3 ^a^	70.18	0.01	0.66	0.10
	Slaughter yield ^§^ (%)	86.85 ^a,b^	89.01 ^a^	87.02 ^a,b^	84.70 ^b^	0.902	0.03	0.01	0.87
	Semi-eviscerated yield ^§^ (%)	79.07	81.54	80.05	78.23	0.981	0.13	0.01	0.85
	Eviscerated yield ^§^ (%)	67.29 ^c^	72.83 ^a^	71.33 ^a,b^	68.82 ^b,c^	1.033	0.01	0.01	0.41
	Abdominal fat yield ^¶^ (%)	0.16 ^c^	0.23 ^b^	0.25 ^a,b^	0.29 ^a^	0.025	0.01	0.08	0.73
	Thigh muscle yield ^¶^ (%)	2.85 ^c^	5.32 ^a^	5.46 ^a^	4.65 ^b^	0.245	0.01	0.02	0.06
	Breast muscle yield ^¶^ (%)	2.66 ^a^	2.45 ^b^	2.36 ^b^	2.30 ^b^	0.064	0.01	0.16	0.84

^a–c^ Within a row, least square means without a common superscript differed (*p* < 0.05). ^†^ CON: control; LMED: low ME diet (9.5 MJ/kg of DM); MMED: medium ME diet (11.5 MJ/kg of DM); HMED: high ME diet (13.5 MJ/kg of DM); Treatment × days on-feed was observed at *p* < 0.05 except for slaughter yield, subcutaneous fat yield, and breast muscle; days on-feed was significant at *p* < 0.05 for all items; * Standard Error of Mean; ^‡^ Treat represents *p*-value of four treatments; Linear and Quadratic represents *p*-value of linear and quadratic change of supplemental feeding treatments (LMED, MMED, and HMED), respectively; ^§^ Calculated as a percentage of live BW; ^¶^ Calculated as a percentage of eviscerated carcass weight.

**Table 5:** Effects of supplemental feeding diets with three levels of metabolizable energy (ME) on meat quality of grazing Swan geese.

Slaughter Day	Meat Quality ^†^	Groups ^‡^				SEM *	*p*-Value ^§^<		
		CON	LMED	MMED	HMED		Treat	Linear	Quadratic
28	L*	53.98 ^a^	39.50 ^b^	39.05 ^b^	38.63 ^b^	1.258	0.01	0.65	0.99
	a*	9.76 ^b^	14.84 ^a^	15.00 ^a^	14.45 ^a^	0.543	0.01	0.51	0.50
	b*	12.42 ^a^	5.03 ^b^	5.08 ^b^	4.82 ^b^	0.319	0.01	0.67	0.71
	Shear force	88.71 ^a^	62.84 ^b^	80.62 ^a^	85.28 ^a^	5.027	0.01	0.02	0.36
56	L*	43.25 ^a^	31.41 ^b^	30.31 ^b^	30.63 ^b^	1.351	0.01	0.16	0.14
	a*	14.44	15.57	15.09	14.99	0.399	0.19	0.23	0.65
	b*	6.53 ^a^	3.60 ^b^	3.27 ^b^	3.28 ^b^	0.375	0.01	0.20	0.42
	Shear force	73.28 ^a^	50.16 ^b^	39.13 ^c^	42.16 ^b,c^	3.028	0.01	0.06	0.16

^a–c^ Within a row, least square means without a common superscript differed (*p* < 0.05). ^†^ L*: lightness; a*: redness; b*: yellowness; * Standard Error of Mean; ^‡^ CON: control; LMED: low ME diet (9.5 MJ/kg of DM); MMED: medium ME diet (11.5 MJ/kg of DM); HMED: high ME diet (13.5 MJ/kg of DM); effect of days on-feed was observed at *p* < 0.01 for L*, a*, b*, and shear force; treatment × days on-feed was observed at *p* < 0.01 except for L*; ^§^ Treat represents *p*-value of four treatments; Linear and Quadratic represents *p*-value of linear and quadratic change of supplemental feeding treatments (LMED, MMED, and HMED), respectively.

**Table 6:** Effects of supplemental feeding diets with three levels of metabolizable energy (ME) on length of section of digestive tract of grazing Swan geese.

Slaughter Day	Section (cm)	Groups ^†^				SEM ^§^	*p*-Value ^‡^<		
		CON	LMED	MMED	HMED		Treat	Linear	Quadratic
0	duodenum	29.00	29.01	28.84	29.06	2.111	0.89	0.94	0.76
	jejunoileal	81.35	78.21	85.28	88.80	4.518	0.27	0.03	0.64
	cecum	18.40	16.81	15.81	16.25	0.874	0.18	0.61	0.44
	rectum	10.57	10.29	10.83	11.06	0.457	0.71	0.27	0.79
28	duodenum	29.13	32.77	33.97	31.53	1.580	0.11	0.51	0.27
	jejunoileal	135.20	139.58	136.90	132.73	6.528	0.68	0.23	0.88
	cecum	21.57	18.70	20.07	19.73	1.203	0.25	0.42	0.45
	rectum	10.57	11.57	11.97	12.60	0.793	0.35	0.36	0.90
56	duodenum	33.35	34.43	34.71	35.49	1.794	0.86	0.71	0.92
	jejunoileal	169.36 ^a^	149.21 ^b^	145.12 ^b^	148.97 ^b^	6.417	0.03	0.97	0.51
	cecum	22.67	21.42	22.93	21.73	0.942	0.53	0.77	0.16
	rectum	13.25	11.72	11.88	12.08	1.049	0.67	0.79	0.98

^a,b^ Least square means within a row with different superscripts differ (*p* < 0.05). ^†^ CON: control; LMED: low ME diet (9.5 MJ/kg of DM); MMED: medium ME diet (11.5 MJ/kg of DM); HMED: high ME diet (13.5 MJ/kg of DM); treatment × days on-feed was observed at *p* < 0.05 for jejunoileal on day 56, but days on-feed was significant at *p* < 0.03 for all items; ^§^ Standard Error of Mean; ^‡^ Treat represents *p*-value of four treatments; Linear and Quadratic represents *p*-value of linear and quadratic change of supplemental feeding treatments (LMED, MMED, and HMED), respectively.

**Table 7:** Effects of supplemental feeding diets with three levels of metabolizable energy (ME) on relative weights of viscera (%) of grazing geese.

Slaughter Day	Viscera (%)	Groups ^†^				SEM ^§^	*p-Value*^‡^<		
		CON	LMED	MMED	HMED		Treat	Linear	Quadratic
0	Heart	0.79	0.77	0.75	0.74	0.030	0.28	0.28	0.83
	Liver	2.82	2.73	2.64	2.64	0.133	0.34	0.38	0.68
	Kidney	0.91	0.90	0.84	0.86	0.036	0.34	0.36	0.27
	Lung	101	0.99	0.92	1.03	0.061	0.38	0.59	0.20
	Spleen	0.062	0.063	0.065	0.067	0.004	0.90	0.54	1.00
	Pancreas	0.42	0.42	0.42	0.43	0.018	0.97	0.87	0.65
	Gizzard	6.63	6.31	6.28	6.47	0.227	0.33	0.33	0.45
	Proventriculus	0.52	0.53	0.50	0.54	0.035	0. 88	0.73	0.44
28	Heart	0.70	0.71	0.79	0.78	0.079	0.69	0.44	0.53
	Liver	2.55 ^b^	3.60 ^a^	3.28 ^a^	2.53 ^b^	0.154	0.01	0.01	0.27
	Kidney	0.57	0.57	0.54	0.58	0.036	0.86	0.86	0.47
	Lung	1.05	1.14	1.37	1.20	0.093	0.13	0.72	0.13
	Spleen	0.097	0.077	0.080	0.085	0.009	0.43	0.48	0.93
	Pancreas	0.53 ^a^	0.40 ^b^	0.36 ^b^	0.38 ^b^	0.027	0.01	0.58	0.27
	Gizzard	7.83 ^a^	4.23 ^b^	3.93 ^b^	4.06 ^b^	0.151	0.01	0.11	0.03
	Proventriculus	1.62 ^a^	0.86 ^b^	0.77 ^b^	0.67 ^b^	0.176	0.01	0.02	0.93
56	Heart	0.61	0.67	0.68	0.73	0.045	0.33	0.36	0.70
	Liver	2.71 ^a^	2.28 ^b^	2.22 ^b^	2.03 ^b^	0.089	0.01	0.04	0.44
	Kidney	0.63 ^a^	0.48 ^b^	0.51 ^b^	0.51 ^b^	0.026	0.01	0.48	0.56
	Lung	1.11 ^a^	0.96 ^b^	0.85 ^b^	0.85 ^b^	0.051	0.01	0.05	0.21
	Spleen	0.062	0.063	0.057	0.057	0.0064	0.83	0.48	0.68
	Pancreas	0.47 ^a^	0.33 ^b^	0.32 ^b^	0.23 ^c^	0.024	0.01	0.01	0.08
	Gizzard	6.20 ^a^	3.61 ^b^	3.37 ^b^	3.13 ^b^	0.159	0.01	0.05	0.99
	Proventriculus	1.02 ^a^	0.61 ^b^	0.60 ^b^	0.58 ^b^	0.105	0.02	0.67	0.99

^a–c^ Within a row, least square means without a common superscript differed (*p* < 0.05); ^†^ CON: control; LMED: low ME diet (9.5 MJ/kg of DM); MMED: medium ME diet (11.5 MJ/kg of DM); HMED: high ME diet (13.5 MJ/kg of DM); Treatment × days on-feed was observed at *p* < 0.01 for liver, lung, pancreas, gizzard, and proventriculus; days on-feed was significant at *p* < 0.05 for all viscera; ^§^ Standard Error of Mean; ^‡^ Treat represents *p*-value of four treatments; Linear and Quadratic represents *p*-value of linear and quadratic change of supplemental feeding treatments (LMED, MMED, and HMED), respectively.

**Table 8:** Effects of supplemental feeding diets with three levels of metabolizable energy (ME) on serum blood biochemical parameters of grazing geese.

Slaughter Day	Blood Metabolites ^†^	Groups ^‡^				SEM *	*p*-Value ^§^ <		
		CON	LMED	MMED	HMED		Treat	Linear	Quadratic
0	ALB (g/L)	7.96	7.96	8.03	7.97	0.49	0.82	0.91	0.36
	GLO (g/L)	31.06	31.48	31.85	27.69	1.42	0.18	0.07	0.20
	TP (g/L)	38.23	39.36	38.72	37.70	1.83	0.83	0.40	0.91
	TG (mmol/L)	0.61	0.57	0.64	0.60	0.02	0.30	0.47	0.11
	TC (mmol/L)	4.59	4.59	4.96	4.69	0.20	0.29	0.66	0.14
	BUN (mmol/L)	0.58	0.60	0.57	0.58	0.03	0.98	0.75	0.73
	Glucose (mmol/L)	5.08	5.43	5.81	5.46	0.20	0.13	0.90	0.16
	ALP (IU/L)	425.3	430.4	438.0	442.8	16.4	0.88	0.61	0.95
28	ALB (g/L)	5.73 ^a^	5.68 ^a^	5.15 ^a,b^	4.48 ^b^	0.37	0.05	0.03	0.86
	GLO (g/L)	23.36 ^ab^	23.77 ^a^	20.99 ^b,c^	18.59 ^c^	1.33	0.01	0.01	0.86
	TP (g/L)	29.09 ^a^	29.45 ^a^	26.14 ^a,b^	23.07 ^b^	1.67	0.01	0.01	0.94
	TG (mmol/L)	0.72 ^b,c^	1.49 ^a^	1.13 ^a,b^	0.66 ^c^	0.16	0.01	0.01	0.77
	TC (mmol/L)	2.96 ^a^	2.13 ^b^	1.93 ^b,c^	1.60 ^c^	0.16	0.01	0.02	0.70
	BUN (mmol/L)	0.46 ^b^	0.89 ^a^	0.47 ^b^	0.69 ^ab^	0.10	0.02	0.21	0.03
	Glucose (mmol/L)	7.89	8.32	7.33	8.03	0.58	0.47	0.55	0.06
	ALP (IU/L)	459.7 ^b^	817.8 ^a^	761.7 ^a^	574.8 ^b^	71.9	0.01	0.04	0.47
56	ALB (g/L)	6.32 ^c^	7.53 ^b^	8.42 ^a,b^	9.23 ^a^	0.36	0.01	0.01	0.95
	GLO (g/L)	25.88 ^c^	29.64 ^b,c^	33.15 ^a,b^	34.71 ^a^	1.34	0.01	0.02	0.58
	TP (g/L)	32.20 ^c^	37.18 ^b^	41.57 ^a^	43.94 ^a^	1.69	0.01	0.02	0.65
	TG (mmol/L)	0.90	1.09	1.00	1.11	0.14	0.67	0.91	0.60
	TC (mmol/L)	2.54	3.10	3.44	3.42	0.30	0.15	0.51	0.67
	BUN (mmol/L)	0.43 ^b^	0.43 ^b^	0.84 ^a^	0.83 ^a^	0.12	0.03	0.07	0.24
	Glucose (mmol/L)	7.62 ^c^	8.12 ^b^	8.90 ^a^	9.35 ^a^	0.22	0.01	0.01	0.58
	ALP (IU/L)	601.5	485.9	570.1	621.3	55.2	0.35	0.12	0.82

^a–c^ Within a row, least square means without a common superscript differed (*p* < 0.05). ^†^ ALB: Albumin; GLO: Globulin; TP: Total protein; TG: Triglyceride; TC: Total cholesterol; BUN: blood urea N; ALP: Alkaline phosphatase; ^‡^ CON: control; LMED: low ME diet (9.5 MJ/kg of DM); MMED: medium ME diet (11.5 MJ/kg of DM); HMED: high ME diet (13.5 MJ/kg of DM); * Standard Error of Mean; ^§^ Treat represents *p*-value of four treatments; Linear and Quadratic represents *p*-value of linear and quadratic change of supplemental feeding treatments (LMED, MMED, and HMED), respectively.

## Data Availability

The data presented in this study are available from the corresponding author on reasonable request.
